# Assessment of Tennessee’s county-level vulnerability to hepatitis C virus and HIV outbreaks using socioeconomic, healthcare, and substance use indicators

**DOI:** 10.1371/journal.pone.0270891

**Published:** 2022-08-04

**Authors:** Jessica Vakili, Lindsey Sizemore, Peter F. Rebeiro, Ben Tyndall, Pamela Talley, Kristyn Whaley, Meredith Brantley

**Affiliations:** 1 Tennessee Department of Health, Nashville, TN, United States of America; 2 Vanderbilt University School of Medicine, Departments of Medicine and Biostatistics, Nashville, TN, United States of America; University of Cincinnati College of Medicine, UNITED STATES

## Abstract

**Background:**

Human immunodeficiency virus (HIV), hepatitis C virus (HCV), and injection drug use are syndemic in the central Appalachian states. In Tennessee (TN), declines in HIV among persons who inject drugs (PWID) stalled, and HCV infection rates increased significantly from 2013–2017. To better target strategies to address the syndemic, county-level socioeconomic, opioid use, access to healthcare, and health factors were modeled to identify indicators predictive of vulnerability to an HIV/HCV outbreak among PWID in TN.

**Methods:**

Newly reported chronic HCV cases among persons aged 13–39 years in 2016–2017 were used as a proxy for county-level HIV/HCV vulnerability among TN’s 95 counties. Seventy-five publicly available county-level measures from 2016–2017 were collected and reduced through multiple dimension reduction techniques. Negative binomial regression identified indicators associated with HCV which were used to calculate county-level vulnerability to a local HIV/HCV outbreak.

**Results:**

Thirteen county-level indicators were identified as strongly predictive of HIV/HCV vulnerability with the statistically significant indicators being percentage of the population aged 20–44 years, per capita income, teen birth rate, percentage of clients in TDMHSAS-funded opioid treatment and recovery, syphilis case rate, and percentage of homes with at least one vehicle. Based on the 13 indicators, we identified the distribution of vulnerability to an HIV/HCV outbreak among TN’s counties. Eleven high vulnerability counties were identified, with the preponderance located in east and middle TN.

**Conclusion:**

This analysis identified the county-level factors most associated with vulnerability to an HIV/HCV outbreak among PWID in TN. These results, alongside routine surveillance, will guide targeted prevention and linkage to care efforts for the most vulnerable communities.

## Introduction

In the United States, high levels of opioid prescribing have led to prevalent misuse of prescription and non-prescription opioids [1]. Opioid-related overdoses accounted for more than 42,000 deaths in 2016, of which 40% were prescription opioids, prompting the United States Department of Health and Human Services to declare the opioid crisis a public health emergency in 2017 [1]. Despite successful efforts to reduce opioid prescribing rates, increasing drug overdose deaths may be a sign of changing drug-related behaviors, such as increases in injection drug use (IDU) and/or the types of drugs being injected. Tennessee (TN) had the third highest opioid prescribing rate in the country in 2017 (94.4 opioid prescriptions for every 100 persons) and there were more than 1,200 deaths (19.3 per 100,000 persons) involving opioids, exceeding the national rate of 14.6 deaths per 100,000 persons during the same time period [1]. As a result, TN is experiencing some of the highest rates of the downstream infectious consequences of the opioid epidemic, including hepatitis C virus (HCV) infection among persons who inject drugs (PWID), and those less than 30 years of age, white, and living in rural areas. Coupled with significant increases in opioid-related overdose deaths over the past five years, these HCV trends underscore the potential for rapid transmission of HIV and/or HCV among PWID.

The overlapping injection drug use, HIV, and HCV epidemics, also known as a syndemic, are the result of structural forces that exacerbate the multiple negative health outcomes among vulnerable populations [2]. HIV and HCV can each be spread through exposure to infected blood, and due to these shared modes of transmission, approximately 75% of persons who inject drugs (PWID) with HIV are co-infected with HCV given, IDU is a known risk factor for both HIV and HCV transmission [3].

Central Appalachia, including TN, is at the center of the injection drug use, HIV, and HCV syndemic. While HIV diagnoses have remained stable in the United States, Southern states accounted for over half of the newly diagnosed HIV cases in 2017 and TN had the 17^th^ highest rate of newly diagnosed HIV nationally; 6.4% of those were attributable to self-reported IDU [4]. According to the Centers for Disease Control and Prevention (CDC) Viral Hepatitis Surveillance Data, the number of reported acute HCV cases increased every year since 2013 and TN ranked 6th highest nationally for acute HCV in 2017, double the national rate [4]. Also, in TN the rate of opioid overdose deaths continued to increase from 11.7 per 100,000 residents in 2013 to 19.3 per 100,000 residents in 2017, with the majority involving multiple contributing drugs [5].

As evidenced by recent HIV outbreaks in the United States, high rates of HCV can be indicative of drug use transmission networks and often precede HIV infection [6, 7]. In 2015, to better understand these trends, the CDC developed a national vulnerability index to identify the counties at highest risk for a rapid HIV and/or HCV transmission event among PWID. Vulnerability extended across the Central Appalachia area with more than 50% of the at-risk counties from the CDC vulnerability assessment located in Kentucky, West Virginia, and TN [6]; 41 of 220 counties were identified in TN [6]. This prompted states to consider completing their own local vulnerability assessment and as a complement to the CDC analysis, in 2016, the TN health department developed an enhanced approach to assessing in-state vulnerability [7]. The TN state health department methodology included an expanded list of 78 indicators including all 15 variables from the CDC analysis as well as locally available data [6, 7]. Findings reinforced that eastern TN was at the highest risk; however, vulnerability was identified throughout the entire state including previously unidentified counties in the rural western corridor.

Targeted and effective public health responses to this evolving syndemic require routine assessments of vulnerability to rapid transmission of HIV and/or HCV infection among PWID. Thus, in 2019, the TN state health department sought to examine updated and newly available data on socioeconomic factors, indicators of healthcare access and opioid use, and select health outcomes and to identify indicators of vulnerability to an HIV/HCV outbreak in TN. As a result, these findings would be used to update county-level vulnerability for earlier detection and response to rapid HIV and HCV transmission among PWID.

## Methods

### Study population

Our study population included TN residents during 2016–2017, aggregated to the county level in all 95 TN counties. In 2017, TN was home to 6.7 million people [8]. TN is in the Southeastern US and is unique in that it borders eight other states; 7 of those states are considered a part of the central Appalachia region [9]. Specifically for TN, the northeastern and eastern counties are part of the Appalachian region. In 2017, the median age in TN was 39 years, 74% of the population in TN was non-Hispanic White, followed by 17% Non-Hispanic Black and 6% Hispanic [8]. The overall poverty rate in TN in 2017 was 15%; however, the highest rates of poverty are concentrated in rural TN, including Northeast and West TN [8].

### Outcome and risk factors

Our outcome variable was defined as hepatitis C cases reported to the National Notifiable Disease Surveillance System using the 2016 chronic HCV case definition, per the Centers of Disease Control and Prevention [10]. Laboratory criterion for diagnosis included a positive test for antibodies to HCV and a positive nucleic acid amplification test (NAAT) for HCV RNA [10]. While the laboratory criterion for acute and chronic HCV are the same, the chronic HCV case definition for HCV does not require clinical criteria [10]. We used the proportion of newly reported chronic HCV cases per 100,000 residents of each county, among those aged 13–39 years in 2016–2017 as our outcome. As the majority of persons with acute HCV infection are asymptomatic (70–85%), the rate of individuals less than 40 years of age with newly reported chronic HCV diagnosis can serve as a proxy for incident cases related to IDU as most newly reported chronic HCV cases in the younger population are the result of injection drug use [11–13]. During 2013–2017, chronic HCV infection rates increased more than 400% in TN and 41% occurred in individuals less than 40 years of age [14].

Seventy-five county level indicators potentially associated with HIV/HCV vulnerability were collected from publicly available data sources (S1 Table), largely informed by the first 2016 in-state vulnerability assessment [7]. In response to the rapidly changing syndemic, our updated assessment incorporated newly available data into an enhanced regression methodology. Data from 2016 and 2017 were collected for each variable and averaged together to create a composite value if both years were available. The majority of indicators (N = 49) came from national sources, including the American Community Survey, County Health Rankings, Substance Abuse and Mental Health Services Administration, the Drug Enforcement Administration, and the National Center for Health Statistics [8, 15–18]. The remaining 26 indicators came from organizations within the state of TN, including the TN Department of Health, TN Bureau of Investigation, TN Department of Transportation, and the TN Department of Mental Health and Substance Abuse Services (TDMHSAS) [19–22]. One variable, the rate of buprenorphine providers per 100,000 population per county, had missing data. To account for the missing data, we created an interaction term using a missing indicator variable multiplied by the rate. The interaction term and original rate variable were both used during dimension reduction. Continuous data that exhibited skewness were natural-log transformed to improve model fit.

### Dimension reduction

**Fig 1:**
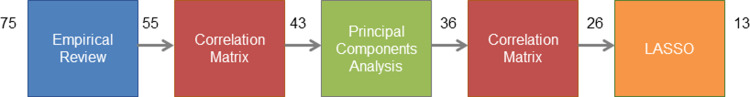
Dimension reduction steps, with number of variables started with and retained at each stage, Tennessee HIV/hepatitis C virus vulnerability assessment—2016–2017 data.

Due to the large number of indicators (N = 75) and small sample size (N = 95), we applied a series of dimension reduction techniques to determine the final set of indicators potentially associated with HIV/HCV vulnerability (Fig 1). Our dimension reduction technique used a combination of subject matter expertise and statistical evidence to retain variables that were data driven but also actionable and meaningful for our state. Our initial variable selection step was an empirical review by a multidisciplinary project team, resulting in the elimination of 20 variables thought to be uninformative for public health action or identifying high-risk populations. Variables with little inter-county variation (less than 14%), better explained by another source, or those that had extensive similarities to other variables were considered and removed. A correlation matrix was used to visualize statistically significant correlations, p-value less than or equal to 0.5, between remaining variables. Twelve indicators that exhibited strong correlation, greater than the absolute value of 0.65, with other variables were subsequently eliminated. Next, principal components analysis was used to identify additional strongly correlated variables, yielding eight components that explained 70% of the model’s variance. This step resulted in the removal of seven variables based on the magnitude of their loadings, a value describing each variables’ contribution to a particular component. Large loadings, either positive or negative, show the strength of correlation between that variable and component. Variables with loadings greater than the absolute value of 0.5 were retained; this cut-off was chosen as it was the mean value and therefore neither too conservative nor too permissive in this variable selection stage. A second correlation matrix was generated and visualized, resulting in the removal of ten additional variables. The final dimension reduction step placed the remaining 26 variables into a Least Absolute Shrinkage and Selection Operator (LASSO) model [23]. LASSO regression, a penalized regression and variable selection method, identified the final 13 data elements used to predict the rate of chronic HCV infections across all TN counties.

### Statistical analysis

Due to overdispersion in the outcome variable, we used negative binomial regression to quantify the association between the final selected indicators and county-level vulnerability to an HIV/HCV outbreak. Indicator coefficients from the multivariable negative binomial model were used in a CDC-authored algorithm to calculate a vulnerability score for each county [6]. To account for uncertainty in the scores, we bootstrapped them using 10,000 samples from a normal distribution for each regression coefficient. County scores were aggregated and ranked from highest to lowest, with higher scores indicating greater vulnerability. Using Tableau’s k-means clustering algorithm, county scores were clustered into three pre-defined categories of vulnerability meaningful for program planning (highly vulnerable, moderately vulnerable, or vulnerable) (Tableau Desktop, v. 2019.3) to produce a county-level map displaying the distribution of vulnerability to an HIV/HCV outbreak across TN. Data manipulation, principal components analysis, and bootstrapping were conducted using SAS v. 9.4 (SAS Institute Inc., Cary, NC). Correlation matrices and negative binomial regression were conducted using R Studio Version 1.2.1335. This analysis was performed as part of a routine program evaluation using public health surveillance data and publicly available data, therefore not submitted to an IRB.

### Dissemination of findings

Two-page informational county-profile documents were created for local community leaders, program planners, and policymakers.

## Results

Thirteen county-level indicators were identified as predictive of vulnerability to an HIV/HCV outbreak among PWID in TN (Table 1). Of the 13 indicators, four were socioeconomic factors: percentage of homes with at least one vehicle, percentage of the population aged 20–44 years, percentage of the population unemployed, and per-capita income. Two indicators were related to drug use: multiple provider episodes (i.e., “doctor shopping”) and morphine milligram equivalent (MME) for all dispensed opioids prescribed for pain. Three variables were related to access to healthcare: primary care provider rate (number of primary providers per 100,000 population), mental health provider rate (number of mental health providers per 100,000 population), and the percentage of clients in TDMHSAS-funded opioid treatment and recovery. The remaining four variables were health outcomes: syphilis case rate (number of syphilis cases per 100,000 residents), teen birth rate (number per 1,000 births among female teens), number of premature deaths (based on years of potential life lost before age 75 per 100,000), and proportion of persons living with HIV (number of living diagnosed HIV cases per 100,000 population). pain. Three variables were related to access to healthcare: primary care provider rate (number of primary providers per 100,000 population), mental health provider rate (number of mental health providers per 100,000 population), and the percentage of clients in TDMHSAS-funded opioid treatment and recovery. The remaining four variables were health outcomes: syphilis case rate (number of syphilis cases per 100,000 residents), teen birth rate (number per 1,000 births among female teens), number of premature deaths (based on years of potential life lost before age 75 per 100,000), and proportion of persons living with HIV (number of living diagnosed HIV cases per 100,000 population).

**Table 1:** Coefficient estimates, standard errors, and p-values from multivariable negative binomial regression model, Tennessee HIV/hepatitis C virus vulnerability assessment—2016–2017 data.

Regression Model Indicator	County-level Indicator Description and Data Source	Coefficient Estimate (95% CI)	Standard Error	P-Value
**Intercept**		2.49 (-0.55,5.58)	1.48	0.09
**Percentage of homes with at least one vehicle**	The number of households with at least one vehicle available divided by the total estimated number of households. Source: American Community Survey, 2016–2017^8^	-2.00 (-4.09, 0.017)	1.01	0.05
**Percentage of population aged 20–44 years**	The number of persons aged 20–44 years divided by the estimated total population. Source: American Community Survey, 2016–2017^8^	5.56 (2.79, 8.35)	1.38	<0.001
**Per capita income**	The mean income per person in the county; derived by dividing the total income of all people by the total population. Source: American Community Survey, 2016–2017^8^	-0.00005 (-0.00008, -0.00001)	0.00002	0.006
**Percentage of the population unemployed**	The number of civilian persons unemployed and actively seeking work divided by the estimated total civilian population aged 16 years and older. Source: American Community Survey, 2016–2017^8^	-8.90 (-18.1, 0.35)	4.74	0.06
**Teen birth rate**	Number of births among female teenagers, 15–19 years old, per 1,000. Source: American Community Survey, 2016–2017^8^	0.0026 (0.0006, 0.0048)	0.001	0.01
**Premature deaths**	A count of the premature deaths that occurred with a county, based on years of potential life lost before age 75 per 100,000 population (age-adjusted). Source: County Health Rankings, 2018^15^	-0.0001 (-0.0003, 0.00005)	0.00008	0.17
**Primary care provider rate**	The number of primary care providers per 100,000 population. Source: County Health Rankings, 2018^15^	0.0021 (-0.0027, 0.0069)	0.002	0.38
**Mental health provider rate**	The number of mental health providers per 100,000 population. Source: County Health Rankings, 2018^19^	-0.0007 (-0.0026, 0.0012)	0.0001	0.45
**Morphine milligram equivalent (MME), log**	The log of the total morphine milligram equivalent for all dispensed opioids prescribed for pain. Source: TN Department of Health Prescription Drug Overdose Program, 2016–2017^19^	0.058 (-0.015, 0.026)	0.104	0.57
**Multiple provider episodes**	Also known as “doctor shopping,” defined as a single patient filling an opioid prescription with at least 5 distinct pharmacies and from at least 5 distinct prescribers in a 6 month period (January 1–June 30 or July 1–December 31). Source: TN Department of Health Prescription Drug Overdose Program, 2016–2017^19^	-0.002 (-0.0054, 0.0015)	0.002	0.28
**Percentage of clients in TDMHSAS-funded opioid treatment and recovery**	Percent of individuals aged 12 years or older receiving Tennessee Department of Mental Health and Substance Abuse Services (TDMHSAS) funded substance abuse treatment and recovery services for any opioid abuse. Source: TDMHSAS, 2017^19^	3.02 (2.34, 3.70)	0.35	<0.001
**HIV prevalence rate**	The number of living diagnosed HIV cases, per 100,000 population. Source: TDH HIV Surveillance Program, 2016–2017^19^	0.0007 (-0.0002, 0.0017)	0.0005	0.13
**Syphilis case rate**	The number of syphilis (primary, secondary, early and late latent) cases per 100,000 population. Source: TDH STD Prevention Program, 2016–2017^19^	0.027 (0.018, 0.037)	0.006	<0.001

Six indicators were associated with chronic HCV infection rate at the county level: percentage of homes with at least one vehicle, percentage of the county aged 20–44 years, per-capita income, teen birth rate, percentage of clients in TDMHSAS-funded opioid treatment and recovery, and syphilis case rate (Table 1). Higher per-capita income and percentage of homes with at least one vehicle were associated with lower rates of chronic HCV (-0.00005 (95% CI: -0.00008, -0.00001), and -2.00 (95% CI: -4.09, 0.017) respectively). Percentage of the county aged 20–44 years and percentage of clients in TDMHSAS-funded opioid treatment and recovery had large, positive coefficients (5.56 (95% CI: 2.79, 8.35) and 3.02 (95% CI: 2.34, 3.7) respectively), and therefore increases in these indicators were related to increased probabilities of chronic HCV infection rate. The coefficients for teen birth rate and syphilis case rate were both close to null (0.0026 (95% CI: 0.0006,0.0048) and 0.027 (0.018,0.0370) respectively), indicating a minimal increase in a county’s chronic HCV infection rate associated with increases in these indicators.

**Fig 2:**
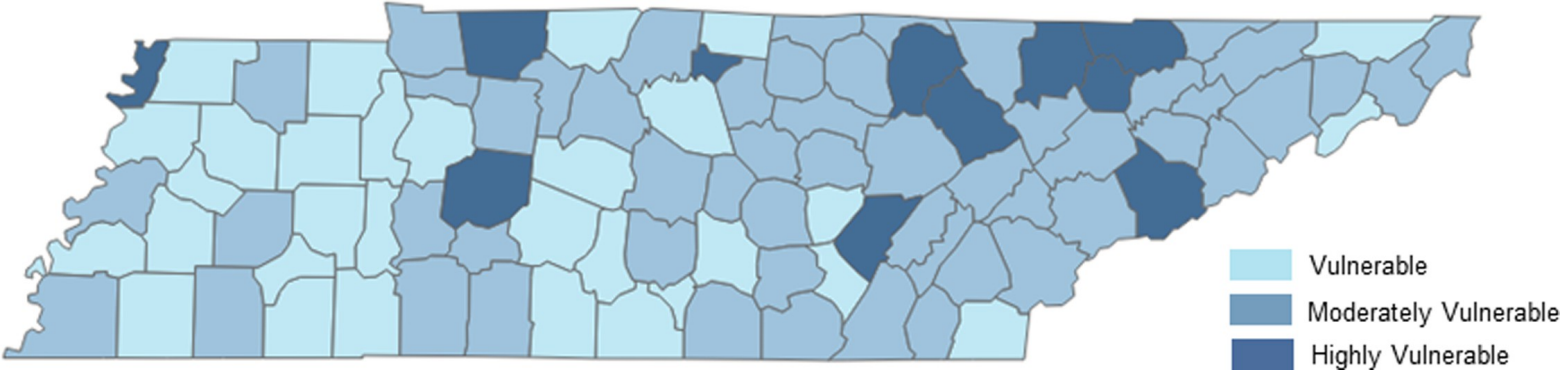
County-level classifications, Tennessee HIV/hepatitis C virus vulnerability assessment—2016–2017 data.

County vulnerability scores ranged from 7.6 to 11.5. Highly vulnerable counties had scores 10.0 and above, counties were classified as moderately vulnerable with scores between 9.0 and 9.9, and vulnerable counties scores were all below 8.9. Eleven highly vulnerable counties were identified across the state, with five located in east TN, four in middle TN, one in southeast TN, and one in west TN (Fig 2). The majority of counties in east TN ranked as highly or moderately vulnerable with six and 24 counties, respectively. In middle TN, four counties were highly vulnerable, 24 were moderately vulnerable, and 13 were vulnerable. In west TN there was one highly vulnerable county, five moderately vulnerable counties, and 15 vulnerable counties.

East TN counties had higher MME values and percentage of clients in TDMHSAS-funded opioid treatment and recovery. Seven of the 11 highly vulnerable counties had a high percentage (ranging from 68%-88%) of individuals receiving TDMHSAS-funded substance abuse treatment and recovery services for opioid abuse, and six of the 11 highly vulnerable counties ranked among the top 20 counties for highest percentage of 20–44 year olds (ranging from 47% to 62%). Additionally, the top 3 ranked counties had low primary care provider rates (ranging from 0% to 9%). Bledsoe County, the most vulnerable county, had the second highest syphilis rate in the state (66.6 cases per 100,000 residents), and Lake County, ranked second in vulnerability, had the highest HIV prevalence rate (787.5 cases per 100,000 population).

## Discussion

TN’s updated vulnerability assessment augments and updates previous work to identify counties vulnerable to the rapid dissemination of HIV or HCV due to IDU [6, 7]. Seven new indicators were identified, and we used six indicators that were included in the 2016 assessment. Of the 11 highly vulnerable counties identified, seven were not included in the top rankings for the 2016 assessment. Five of the six metropolitan counties in TN, previously classified as low vulnerability jurisdictions, emerged as moderately vulnerable. The most vulnerable counties were clustered throughout the east and northeastern regions of the state, with additional vulnerability found in select counties of middle and western TN. High vulnerability was concentrated in rural areas comprised of younger communities with disproportionate rates of unemployment and high rates of HIV, sexually transmitted infections, HCV, and opioid use with less access to primary and behavioral health care services. Results highlight the syndemic nature of socioeconomic factors, opioid use, health care access, and health outcomes that collectively contribute to conditions ripe for an HIV or HCV outbreak among PWID.

Our assessment focused on the risk of rapid transmission of HIV or HCV among persons who inject drugs (PWID); however, transmission networks are rarely homogeneous as PWID may also engage in sexual activity that introduces risk for HIV or HCV [24]. Advancements in HIV surveillance have elucidated local transmission dynamics. During recent cluster investigations in Massachusetts, West Virginia, and TN, it was determined that local transmission may be driven by overlapping networks of drug and sexual risk behavior (e.g., IDU, male-to-male sexual contact, and high-risk heterosexual contact) [25–27], demonstrating a need for innovative, multipronged approaches to reach and serve all communities at risk. Maximum prevention impact will be achieved by leveraging resources across sectors to address multiple health (HIV, HCV, substance use disorder) and socioeconomic support (housing, transportation, employment) needs of vulnerable individuals.

Federal and state resources are available to support routine HIV and HCV surveillance and prevention activities to reduce the spread of HIV and HCV. The findings of this vulnerability assessment highlight the need for additional funding to support implementation of integrated, evidence-based prevention interventions to mitigate the transmission of bloodborne infections related to the opioid epidemic. Evidence-based practices are most effective through approaches that target structural forces, integrate systems of care, and provide comprehensive harm reduction services to clients. Many of the 13 county-level indicators identified can be impacted by public health action through policy and systems level change. Specifically, multiple provider episodes and MME are largely guided by state law regarding use of the Controlled Substance Monitoring Database and clinical guidelines for providers [28]. Moreover, access to primary care and mental health providers is reflective of public and private sector investment in health care delivery. Coupled with expansion of transportation services, implementation or scale up in the use of telehealth programs would enhance access to primary and behavioral health care, particularly in rural settings facing hospital closures and provider shortages [29, 30]. Support services to address housing, employment, criminal justice, and education needs will be critical to effectively serve the most vulnerable communities. Agencies that employ persons with lived experience, often referred to as peer navigators, can optimize the delivery of multiple health and social support services [31–33].

The state health department and the TN Department of Mental Health and Substance Abuse Services (TDMHSAS) support the delivery of a variety of client-level harm reduction services. These include 4 syringe services programs (SSPs) and numerous program-specific navigators who specialize in linking PWID to resources for HIV prevention (i.e., pre-exposure prophylaxis [PrEP]), HIV treatment, HCV treatment, overdose prevention (i.e., naloxone), and substance use disorder treatment. SSPs are particularly important in highly vulnerable areas for preventing and curbing HIV and HCV transmission among PWID. However, several counties identified as highly vulnerable do not have local SSPs or related harm reduction programs [34]. Expansion in areas of highest need is critical to reducing HIV/HCV outbreak vulnerability across the state and specifically comprehensive SSPs which are currently concentrated in urban counties leaving substantial unmet need in more rural areas [35–37]. Jurisdictions equipped to deliver comprehensive harm reduction services will help to mitigate local vulnerability to IDU-related outbreaks, while sustaining the capacity to respond quickly to an event [38–40].

Limitations of our approach included insufficient available county-level data on incarceration rates for regression modeling; sensitivity analyses omitting high-imprisonment-rate counties did not reveal a substantial change in relative ranks of counties. Five of the 11 counties ranked most vulnerable contain a prison, which are known to house a population at higher risk for HCV, our outcome variable. Thus, the overall risk for the non-incarcerated population in these counties might be overstated. Starting in 2018, testing incarcerated individuals on intake for HCV became standard practice. Future analyses will explore removing HCV cases found in incarcerated individuals in order to examine the impact on county rankings. Though results reflect population-level inferences and were derived from county-level data, counties are relevant policy-making jurisdictions, and county-level inferences may be informative for funding and health policy decisions within TN. The focus of our analysis was not on the magnitude or precision of individual indicators but on the calibration and performance of the prediction model *in toto*. As tests of statistical significance rest on an assumption of random sampling from the target population, our hypothesis tests may have been biased to the extent that the administrative or surveillance data collected were incomplete or obtained from only a select population within each county; however, given reporting requirements and census practices, we expect these sampling assumptions are largely met. Another limitation and possible source of measurement error in our outcome is the lack of a direct IDU measure; however, the use of proxy measures reflects best practices in related research at both the state and national levels [6, 13]. Due to volume in TN, we do not collect chronic HCV risk factors but TN mirrors national trends between injection drug use and HCV [41]. In addition, although a standard case definition exists for chronic HCV and it is a reportable condition to the TN Department of Health, there may be measurement error due to varying HCV testing and reporting practices across TN. In future work, we plan to explore more proximal measures such as IDU-related soft-tissue infections and endocarditis resulting in hospitalization [42, 43]. Despite these limitations, we believe this work and similar studies provide essential data and relatively unbiased inferences to inform public health action. The use of multiple data sources and methods of developing prediction models provides insight into efficient resource allocation, while describing changing trends in these ongoing epidemics.

Findings should be communicated effectively to non-governmental and governmental stakeholders in order to successfully procure additional resources, while focusing and prioritizing efforts in areas where they are needed most. The two-page county profiles include key HIV, HCV, and drug overdose facts for TN, a brief overview of the assessment, a description of each county’s vulnerability status, a summary table of state-level and county-level indicators, and references to find more information on preventing an HIV or HCV outbreak. The findings were used to support a new public-facing website that displays an interactive map of TN’s county-level vulnerability to a rapid HIV/HCV transmission event, a county profile for each county, relevant state health department and TDMHSAS resources, and a comprehensive statewide plan that focuses on recommendations for action [44].

Collecting, analyzing, and translating local data about the HIV, HCV, and injection drug use syndemic has equipped both public health and community stakeholders with information needed to properly identify and address resource gaps, as well as pursue policy-related changes. Findings will be used to inform strategic planning addressing the syndemic by targeting individual-, community-, and structural-level factors, including those identified through TN’s vulnerability assessment. Moreover, routine monitoring and evaluation of county-level vulnerability in TN will be critical to ensuring resources are responsive to changes in the TN syndemic and are effectively directed to identify, prevent, and respond to rapid HIV/HCV transmission events.

Due to new data and the changing opioid epidemic, TN completed an updated vulnerability assessment in 2019 to inform HIV and Viral Hepatitis Program activities, including placement of harm reduction resources navigators and funding for SSP expansion. Like the previous assessment, our analysis plan utilized programmatic expertise and data driven methods. Our focus in the latest assessment was to prepare a systematic plan that could be easily replicated in future iterations in an effort to monitor the epidemic and populations disproportionately impacted. The latest assessment’s outcome variable was defined as newly chronic HCV in individuals 13–39 years of age versus acute HCV, as used previously. While using chronic HCV has limitations listed previously in the discussion, acute HCV is difficult to diagnose given the lack of symptoms or symptoms that do not prompt an individual to seek medical care and provider reporting may be incomplete. Using the most complete yet current data examines if county-level risk changes as the injection drug use, HCV, and HIV epidemics transform or as other situations arise that impact these results (e.g., hepatitis A virus outbreak, COVID-19 pandemic). Furthering targeted and effective public health responses to the ongoing syndemic require current assessments of vulnerability to rapid transmission of HIV and/or HCV infection among PWID.

## Supporting information

S1 Table Summary of 75 potential HIV/HCV outbreak vulnerability model indicators, description, and data sources—Tennessee, 2016–2017 data. (PDF)

S2 Table Publicly available data used in analysis. Variables listed are the average of 2016 and 2017 data.
(XLS)

S3 Table 21 variables used in analysis that are available upon request with contact information provided. (PDF)
