# Supplementary material for: Assessment of Tennessee’s county-level vulnerability to hepatitis C virus and HIV outbreaks using socioeconomic, healthcare, and substance use indicators
Source: PLoS One. 2022 Aug 4;17(8):e0270891. doi: 10.1371/journal.pone.0270891 (PMC9352017; doi:10.1371/journal.pone.0270891)
Supplement: S1 Table — (PDF) [file pone.0270891.s001.pdf]

Supplementary table 1: summary of 75 potential HIV/HCV outbreak vulnerability model indicators, description, and data sources — Tennessee, 2016-2017 data

Potential Indicator	Indicator Description (County-level)	Data Source
Acute hepatitis C infection rate	Number of acute Hepatitis C infection cases per 100,000 population	TDH Viral Hepatitis Program, 2016–2017 average
Adults reporting poor/fair health	Percentage of adults reporting fair or poor health (age-adjusted).	County Health Rankings, 2018
Behavioral Health Safety Net Service Rate	Rate of individuals receiving Behavioral Health Safety Net Services, an uninsured population. Rate per 1,000 people served in each county	TDMHSAS, 2017
Certified pain management clinics	Count of certified pain clinics per county. This does not capture pain clinics that are not registered.	Health Licensure and Regulation, 2017
Chronic hepatitis C infection rate	Number of chronic hepatitis C infection cases, ages 13-39, per 100,000 population	TDH Viral Hepatitis Program, 2016–2017 average
Crowded housing units	More than 1 person per bedroom for a given residence. This measure is the average percentage of homes that were defined as being crowded.	American Community Survey, 2016–2017 average
Death rate, all drugs	Number of deaths related to all drugs, per 100,000.	TDH Prescription Drug Overdose Program, 2016–2017 average
Death rate, heroin and opioids only	Number of deaths related to heroin and opioids, per 100,000.	TDH Prescription Drug Overdose Program, 2016–2017 average
Deaths related to all drugs	The count of all deaths attributed to any kind of drug. The county of residence for the person who died is captured.	TDH Prescription Drug Overdose Program, 2016–2017 average
Deaths related to heroin and opioids only	The count of all deaths attributed to heroin or opioids. The county of residence for the person who died is captured.	TDH Prescription Drug Overdose Program, 2016–2017 average
Drug coalition	Funded drug coalition present within the county (yes/no).	TDMHSAS, fiscal year 2018
Drug trafficking 'hot zone'	County identified as a 'drug trafficking hot zone' by the department of justice (yes/no).	High Intensity Drug Trafficking Area (HIDTA) Program, 2017
Drug-related crimes	The number of drug crimes (distribution and selling charges, possession and concealing charges) in a county. This includes only narcotic drugs.	Tennessee Bureau of Investigation, 2016–2017 average
Female headed households	Average number of female headed households (divorced, widowed, never married and over 18).	American Community Survey, 2016–2017 average
GINI Coefficient	Summary measure of income inequality. Values range from 0 to 1, with higher scores indicating greater inequality.	American Community Survey, 2016–2017 average
Highway access	At least 1 interstate or major US highway exit in or within 5 miles of the county border.	TN Department of Corrections
HIV cases related to injection drug use	The count of any living diagnosed HIV cases related to injection drug use.	TDH HIV Surveillance Program, 2016–2017 average
HIV incidence rate	The number of new HIV diagnoses, per 100,000 population.	TDH HIV Surveillance Program, 2016–2017 average
HIV prevalence rate	The number of living diagnosed HIV cases, per 100,000 population.	TDH HIV Surveillance Program, 2016–2017 average
Homes with no phone service	Average number of homes without phone service.	American Community Survey, 2016–2017 average
Injury related deaths	Number of deaths related to injuries.	County Health Rankings, 2018
Mental health provider rate	The number of mental health providers per 100,000 population.	County Health Rankings, 2018
Mental health providers	The number of mental health providers.	County Health Rankings, 2018
Methadone clinics	Presence of at least one methadone clinic for a given county (yes/no).	TDMHSAS, 2017
Missing indicator for buprenorphine providers	A missingness indicator was included to address the missingingess of this variable.	SAMHSA
Morphine milligram equivalent (MME), log	The log of the total morphine milligram equivalent for all dispensed opioids prescribed for pain.	TDH Prescription Drug Overdose Program, 2016–2017 average
Multiple provider episodes	Also known as "doctor shopping," defined as a single patient filling an opioid prescription with at least 5 distinct pharmacies and from at least 5 distinct prescribers in a 6 month period (January 1–June 30 or July 1–December 31).	TDH Prescription Drug Overdose Program, 2016–2017 average
National Center for Health Statistics (NCHS) rural-urban classification	Counties were categorized into one of six categories based on delineation of metropolitan statistical areas and micropolitan statistical areas: 1) Large central metro, 2) Large fringe metro, 3) Medium metro, 4) Small metro, 5) Micropolitan, and 6) Non-core.	NCHS, 2013
Neonatal abstinence syndrome cases	The number of neonatal abstinence syndrome cases per county.	Family Health and Wellness, 2016–2017 average
Non-fatal overdose rate, all drugs	Number of non-fatal overdoses, per 100,000 population, that resulted in a hospitalization or emergency department visit regardless of the drug type that caused the overdose.	TDH Prescription Drug Overdose Program, 2016–2017 average
Non-fatal overdose rate, heroin only	Number of non-fatal overdoses that resulted in a hospitalization or emergency department visit for heroin only.	TDH Prescription Drug Overdose Program, 2016–2017 average
Non-fatal overdose rate, opioids only	Number of non-fatal overdoses, per 100,000 population, that resulted in a hospitalization or emergency department visit for opioids only.	TDH Prescription Drug Overdose Program, 2016–2017 average
Number of mobile homes	Number of mobile homes.	American Community Survey, 2016–2017 average
Occupied housing units	Number of occupied housing units.	American Community Survey, 2016–2017 average

Potential Indicator	Indicator Description (County-level)	Data Source
Patients filling buprenorphine prescriptions	The number of people per county filling buprenorphone prescriptions as a way to measure adoption of buprenorphine treatment.	TDH Prescription Drug Overdose Program, 2016–2017 average
Per capita income	Mean income per person in the county; derived by dividing the total income of all people by the total population.	American Community Survey, 2016–2017 average
Per capita income, log	Mean income per person in the county; derived by dividing the total income of all people by the total population and modeled as log base 10.	American Community Survey, 2016–2017 average
Percentage of adults who smoke	Percentage of adults who are current smokers.	County Health Rankings, 2018
Percentage of clients in TDMHSAS-funded opioid treatment and recovery	Percent of individuals aged 12 years or older receiving Tennessee Department of Mental Health and Substance Abuse Services (TDMHSAS) funded substance abuse treatment and recovery services for any opioid abuse.	TDMHSAS, 2017
Percentage of homes with at least one vehicle	Number of households with a vehicle available divided by the total estimated number of households per county.	American Community Survey, 2016–2017 average
Percentage of homes with no phone Service	Average percentage of the total housing units without phone service.	American Community Survey, 2016–2017 average
Percentage of mobile homes	Percentage of the total housing units that were mobile homes, calculated by dividing the total number of mobile homes by the total number of housing units.	American Community Survey, 2016–2017 average
Percentage of population aged 20–24 years	Percentage of those aged 20–24; calculated by dividing the population aged 20-24 by the estimated population.	American Community Survey, 2016–2017 average
Percentage of population aged 20–44 years	Percentage of those aged 20–44; calculated by dividing the population aged 20-44 by the estimated population.	American Community Survey, 2016–2017 average
Percentage of population identified as white non-Hispanic	Number of persons who reported they were not Hispanic or Latino and were of white race alone divided by the estimated total county population.	American Community Survey, 2016–2017 average
Percentage of population living in poverty	Based on US Census Bureau income thresholds by family size and composition. If a family's total income is less than the family's threshold, then that family and every individual in it is considered in poverty. The number of persons in poverty was divided by the estimated total county population.	American Community Survey, 2016–2017 average
Percentage of population never married	The percent of the population of a county that was never married, calculated by dividing the number of residents who were never married by the estimated population.	American Community Survey, 2016–2017 average
Percentage of population unemployed	Number of civilian persons unemployed and actively seeking work divided by the estimated total civilian population aged 16 years and older.	American Community Survey, 2016–2017 average
Percentage of population uninsured	Number of persons without health insurance coverage was divided by total civilian noninstitutionalized population.	American Community Survey, 2016–2017 average
Percentage of population with a disability	Percent of the population with a disability.	American Community Survey, 2016–2017 average
Percentage of population with no high school diploma	Number of persons aged 25 years and older with less than a 12th grade education (including individuals with 12 grades but no diploma) divided by the estimated county population age 25 years and older.	American Community Survey, 2016–2017 average
Poor mental health days	Number of mentally unhealthy days reported in past 30 days (age-adjusted).	County Health Rankings, 2018
Poor physical health days	Number of physically unhealthy days reported in past 30 days (age-adjusted).	County Health Rankings, 2018
Population aged 20–24 years	Average number of people aged 20–24.	American Community Survey, 2016–2017 average
Population decline, 2000–2016	A measure of population decline at the county level between 2000 and 2016.	American Community Survey, 2016–2017 average
Population estimate	Estimated population for each county.	American Community Survey, 2016–2017 average
Population estimate, log	Estimated population for each county, modeled as log base 10.	American Community Survey, 2016–2017 average
Population per square mile	Population per square mile.	American Community Survey, 2016–2017 average
Population per square mile, log	Population per square mile, modeled as log base 10.	American Community Survey, 2016–2017 average
Premature deaths	Count of the premature deaths that occurred with a county, based on years of potential life lost before age 75 per 100,000 population (age-adjusted).	County Health Rankings, 2018
Primary care provider rate	The number of primary care providers per 100,000 population.	County Health Rankings, 2018
Primary care providers	Number of primary care providers.	County Health Rankings, 2018
Rate of buprenorphine providers	Number of buprenorphine providers, per 100,000 population, in each county. Interaction term with missinginess indicator.	SAMHSA
Rate of Injury related deaths	Number of deaths related to injuries per 100,000 population.	County Health Rankings, 2018
Rate of syphilis infections	The number of syphilis (primary, secondary, early and late latent) cases per 100,000 population	TDH STD Prevention Program, 2016–2017 average

Potential Indicator	Indicator Description (County-level)	Data Source
Residents who were never married	The number of people aged 15 and older who report never being married.	American Community Survey, 2016–2017 average
Sexually transmitted disease rate	The rate of sexually transmitted disease (gonorrhea, chlamydia) per 100,000 population by county. 2016 and 2017 data were collected and averaged for each county to calculate this measure.	American Community Survey, 2016–2017 average
Social associations	The number of membership associations.	County Health Rankings, 2018
Social associations eate	The number of membership associations per 10,000 population.	County Health Rankings, 2018
Teen birth rate	Number of births among female teenagers, 15–19 years old, per 1,000.	American Community Survey, 2016–2017 average
Theft crimes	The number of theft crimes (robbery, pickpocketing, purse snatching, shoplifting, from buildings, from coin machines, from motor vehicles, of motor vehicle parts, of motor vehicles) in a county.	Tennessee Bureau of Investigation, 2016–2017 average
Total housing units	Number of housing units.	American Community Survey, 2016–2017 average
Vacant housing units	Number of vacant housing units.	American Community Survey, 2016–2017 average
Violent crimes	The number of violent crimes (murder, forcible rape, aggravated assault) in a county.	Tennessee Bureau of Investigation, 2016–2017 average
Years of potential life lost	Years of potential life lost before age 75 per 100,000 population (age-adjusted).	County Health Rankings, 2018

TDH: Tennessee Department of Health

TDMHSA: Tennessee Department of Mental Health and Substance Abuse Services
