# Supplementary material for: Assessment of Tennessee’s county-level vulnerability to hepatitis C virus and HIV outbreaks using socioeconomic, healthcare, and substance use indicators
Source: PLoS One. 2022 Aug 4;17(8):e0270891. doi: 10.1371/journal.pone.0270891 (PMC9352017; doi:10.1371/journal.pone.0270891)
Supplement: S3 Table — (PDF) [file pone.0270891.s003.pdf]

Supplementary Table 3: 21 variables used in analysis that are available upon request with contact information provided

Variable	Description	Source	Contact Information
Drug Coalition?	Is there a funded drug coalition present within the county? Fiscal year 2018 data were used.	Tennessee Department of Mental Health and Substance Abuse Services	Requested from Tony Jackson, TDMHSAS Director of Prevention Services, anthony.jackson@tn.gov
Certified pain management clinics	The count of certified pain clinics per county. This does not capture pain clinics that are not registered. Data is 2017 and prior.	Health Licensure and Regulation	Requested from Tonya Wilkins Pain Management Clinic Administrator, painmanagement.health@tn.gov
Neonatal Abstinence Syndrome Cases	The number of Neonatal Abstinence Syndrome cases per county. 2016 and 2017 data were collected and averaged for each county.	Family Health and Wellness	Requested from Dr. Dr. Alice Nyakeriga, NAS Epidemiologist, alice.nyakeriga@tn.gov
Rate of HIV Incidence	The rate of new HIV diagnoses, per 100,000 population, by county. 2016 and 2017 data were collected and averaged for each county to calculate this measure.	Enhanced HIV/AIDS Reporting System	Requested from TN Department of Health, https://www.surveygizmo.com/s3/5819792/TDH-Data-Request-Form
Rate of HIV Prevalence	The rate, per 100,000 population, of living diagnosed HIV cases, by county. 2016 and 2017 data were collected and averaged for each county to calculate this measure.	Enhanced HIV/AIDS Reporting System	Requested from TN Department of Health, https://www.surveygizmo.com/s3/5819792/TDH-Data-Request-Form
HIV cases related to Injection Drug Use	The count of any living diagnosed HIV cases related to injection drug use, by county. 2016 and 2017 data were collected and averaged for each county.	Enhanced HIV/AIDS Reporting System	Requested from TN Department of Health, https://www.surveygizmo.com/s3/5819792/TDH-Data-Request-Form
Rate of Sexually Transmitted Diseases	The rate of sexually transmitted disease (gonorrhea, chlamydia) per 100,000 population by county. 2016 and 2017 data were collected	Patient Reporting Investigation	Requested from TN Department of Health, https://www.surveygizmo.com/s3/5819792/TDH-Data-Request-Form

	and averaged for each county to calculate this measure.	Surveillance Manager	
Rate of Syphilis Infections	The rate of syphilis (primary, secondary, early, and late latent) cases per 100,000 residents, by county. 2016 and 2017 data were collected and averaged for each county to calculate this measure.	Patient Reporting Investigation Surveillance Manager	Requested from TN Department of Health, https://www.surveygizmo.com/s3/5819792/TDH-Data-Request-Form
Rate of Acute Hepatitis C Infections	The rate of acute Hepatitis C infection cases per 100,000 residents, by county. 2016 and 2017 data were collected and averaged for each county to calculate this measure.	National Electronic Disease Surveillance System Based System	Requested from TN Department of Health, https://www.surveygizmo.com/s3/5819792/TDH-Data-Request-Form
Rate of Chronic Hepatitis C Infections	The rate of chronic Hepatitis C infection cases, ages 13-39, per 100,000 residents, by county. 2016 and 2017 data were collected and averaged for each county to calculate this measure.	National Electronic Disease Surveillance System Based System	Requested from TN Department of Health, https://www.surveygizmo.com/s3/5819792/TDH-Data-Request-Form
Non-fatal Overdoses, all drugs	The rate of non-fatal overdoses, per 100,000 population, that resulted in a hospitalization or emergency department visit regardless of the drug type that caused the overdose by county. 2016 and 2017 data were collected and averaged for each county to calculate the rate.	Prescription Drug Overdose Program	2017 non-fatal data: https://www.tn.gov/content/tn/health/health-program-areas/pdo/pdo/data-dashboard.html#downloadabledata; 2016 non-fatal data: Requested from TN Department of Health, https://www.surveygizmo.com/s3/5819792/TDH-Data-Request-Form

Non-fatal Overdoses, opioids only	The rate of non-fatal overdoses, per 100,000 population, that resulted in a hospitalization or emergency department visit for opioids only, by county. 2016 and 2017 data were collected and averaged for each county to calculate the rate.	Prescription Drug Overdose Program	2017 non-fatal data: https://www.tn.gov/content/tn/health/health-program-areas/pdo/pdo/data-dashboard.html#downloadabledata; 2016 non-fatal data: Requested from TN Department of Health, https://www.surveygizmo.com/s3/5819792/TDH-Data-Request-Form
Non-fatal Overdoses, heroin only	The rate of non-fatal overdoses that resulted in a hospitalization or emergency department visit for heroin only, by county. 2016 and 2017 data were collected and averaged for each county to calculate the rate.	Prescription Drug Overdose Program	2017 non-fatal data: https://www.tn.gov/content/tn/health/health-program-areas/pdo/pdo/data-dashboard.html#downloadabledata; 2016 non-fatal data: Requested from TN Department of Health, https://www.surveygizmo.com/s3/5819792/TDH-Data-Request-Form
Total MME for all drugs	The total morphine milligram equivalent for all opioids for pain with a conversion factor, per county. 2016 and 2017 data were collected and averaged for each county.	Prescription Drug Overdose Program	Data Request: Requested from TN Department of Health, https://www.surveygizmo.com/s3/5819792/TDH-Data-Request-Form
Log of Total MME for all drugs	The log of the total morphine milligram equivalent for all opioids for pain with a conversion factor, per county. 2016 and 2017 data were collected and averaged for each county.	Prescription Drug Overdose Program	Data Request: Requested from TN Department of Health, https://www.surveygizmo.com/s3/5819792/TDH-Data-Request-Form
The number of patients filling buprenorphine prescriptions	The number of people per county filling buprenorphine prescriptions to measure adoption of buprenorphine treatment. 2016 and 2017 data were collected and averaged for each county.	Prescription Drug Overdose Program	Data Request: Requested from TN Department of Health, https://www.surveygizmo.com/s3/5819792/TDH-Data-Request-Form

Multiple provider Episodes	The count of MPEs that occurred in each county defined as a single patient filling an opioid prescription with at least five distinct pharmacies and from at least five distinct prescribers in a 6-month period (either January 1 - June 30 or July 1 - December 31). An MPE may also occur for a patient who has prescriptions written by multiple providers in a single practice and uses multiple pharmacies. A distinct patient may have a multiple provider episode once in each 6-month period for a total of 2 potential MPEs per year.	Prescription Drug Overdose Program	Data Request: Requested from TN Department of Health, https://www.surveygizmo.com/s3/5819792/TDH-Data-Request-Form
Deaths related to all drugs	The count of all deaths attributed to any kind of drug. The county of residence for the person who died is captured. 2016 and 2017 data were collected and averaged for each county.	Prescription Drug Overdose Program	2017 fatal data: https://www.tn.gov/content/tn/health/health-program-areas/pdo/pdo/data-dashboard.html#downloadabledata; 2016 fatal data: Requested from TN Department of Health, https://www.surveygizmo.com/s3/5819792/TDH-Data-Request-Form
Deaths related to heroin and opioids only	The count of all deaths attributed to heroin or opioids. The county of residence for the person who died is captured. 2016 and 2017 data were collected and averaged for each county.	Prescription Drug Overdose Program	2017 fatal data: https://www.tn.gov/content/tn/health/health-program-areas/pdo/pdo/data-dashboard.html#downloadabledata; 2016 fatal data: Requested from TN Department of Health, https://www.surveygizmo.com/s3/5819792/TDH-Data-Request-Form
Rate of Death, all drugs	The rate, per 100,000, of the number of deaths related to all drugs. 2016 and 2017 data were collected and averaged for each county.	Prescription Drug Overdose Program	2017 fatal data: https://www.tn.gov/content/tn/health/health-program-areas/pdo/pdo/data-dashboard.html#downloadabledata; 2016 fatal data: Requested from TN Department of Health, https://www.surveygizmo.com/s3/5819792/TDH-Data-Request-Form

Rate of Death, heroin, and opioids only	The rate, per 100,000, of the number of deaths related to heroin and opioids. 2016 and 2017 data were collected and averaged for each county.	Prescription Drug Overdose Program	2017 fatal data: https://www.tn.gov/content/tn/health/health-program-areas/pdo/pdo/data-dashboard.html#downloadabledata; 2016 fatal data: Requested from TN Department of Health, https://www.surveygizmo.com/s3/5819792/TDH-Data-Request-Form
